# Case Report of Management of Fowler’s Syndrome During Pregnancy: Challenges and Outcomes

**DOI:** 10.7759/cureus.79523

**Published:** 2025-02-23

**Authors:** Rodrigo F Rodrigues, Marcelo Antonini, Maria Luiza Toledo, Eliza K Silva, Hudson F Silva

**Affiliations:** 1 Obstetrics and Gynaecology Department, Servidor Público Estadual Hospital – Francisco Morato Oliveira (HSPE-FMO), Sao Paulo, BRA; 2 Mastology Department, Servidor Público Estadual Hospital – Francisco Morato Oliveira (HSPE-FMO), Sao Paulo, BRA; 3 Obstetrics Department, Servidor Público Estadual Hospital – Francisco Morato Oliveira (HSPE-FMO), Sao Paulo, BRA

**Keywords:** c-section, fowler's syndrome, itu, obstetric labor, pre-natal care, sacral neuromodulation, urinary retention (ur)

## Abstract

Fowler’s syndrome (FS) is a rare cause of urinary retention affecting young women, characterized by dysfunctional urethral sphincter relaxation. Its management during pregnancy presents challenges, particularly regarding sacral neuromodulation (SNM), which is the preferred treatment but remains controversial due to concerns about uterine contractions and limited safety data. Intermittent catheterization is an alternative but increases the risk of urinary tract infections and pregnancy complications. This case report aims to describe the obstetric management and clinical outcomes of a pregnant patient with Fowler’s syndrome undergoing SNM. A 25-year-old woman with FS conceived unexpectedly and, concerned about potential pregnancy risks, deactivated the device and initiated intermittent catheterization. However, she experienced persistent urinary retention, nocturia, and sciatic pain, prompting her to reactivate the device without medical supervision. Despite these challenges, her pregnancy progressed without urinary tract infections or preterm labor. At 37 weeks and four days, she underwent a planned cesarean section, delivering a healthy newborn. Managing FS during pregnancy is complex due to the risks and benefits of SNM versus intermittent catheterization. This case highlights the need for individualized, multidisciplinary care. Further research is essential to develop standardized guidelines to optimize maternal and fetal outcomes.

## Introduction

First described by Clare Fowler in 1988, Fowler's syndrome (FS) remains an enigmatic condition. It is characterized by sustained urethral sphincter contraction and an inability to relax, predominantly affecting young women and occasionally associated with polycystic ovary syndrome (PCOS) [1]. External stress factors may trigger the condition, with episodes varying in duration from months to years. The pathophysiology is believed to involve abnormal electromyographic activity in the external urethral sphincter, possibly due to dysfunctional communication between the sacral spinal cord and the detrusor muscle. This results in increased sphincter activity, leading to urinary retention, which may be associated with polycystic ovary syndrome and hormonal fluctuations [2].

Diagnosis necessitates the exclusion of neurological and urological disorders and is typically confirmed through urodynamic studies in conjunction with bladder ultrasound (video urodynamic studies). Treatment aims to ensure complete bladder emptying, achieved through intermittent catheterization, surgical diversion, or botulinum toxin injections. Sacral neuromodulation is considered the gold standard [3].

Sacral neuromodulation (SNM) involves the implantation of an electrode along the sacral nerve root, connected to a pulse generator. The patient can deactivate the device via an external electronic control if necessary [4]. Its application during pregnancy remains contentious due to the stimulation of the S3 and S4 nerves, which may provoke uterine contractions. The exact mechanism of action is not fully understood, and theoretical risks of teratogenic effects have led obstetricians and device manufacturers to frequently contraindicate its use [5].

Pregnancy introduces unique challenges in the management of FS, as hormonal and anatomical changes can exacerbate urinary retention or modify the response to SNM therapy. The growing uterus may alter the positioning of the implanted electrode, potentially affecting its efficacy or causing discomfort. Additionally, while some case reports suggest that SNM can be safely continued throughout pregnancy, others have documented complications such as increased pain at the implantation site or a need for device reprogramming [5].

Unlike sacral neuromodulation, intermittent catheterization necessitates appropriate training to ensure aseptic technique. The primary concern during pregnancy is the increased risk of urinary tract infections (UTIs), which can precipitate severe complications such as pyelonephritis, preterm birth, fetal growth restriction, sepsis, and maternal-fetal mortality [6].

The optimal delivery method for patients with sacral neurostimulators remains controversial. During vaginal delivery, the sacral electrode may become displaced, and a cesarean section has been suggested as a way to reduce this risk. However, cesarean delivery has also been associated with a higher incidence of device dysfunction. Some studies recommend cesarean delivery to minimize potential damage to the sacral electrode [3].

Currently, data provide no definitive guidance on whether to deactivate the neurostimulator during pregnancy. Reports indicate variable outcomes, with cases of preterm birth associated with activated devices and instances of pyelonephritis and ICU admissions following deactivation and subsequent self-catheterization. No standardized management protocol exists for pregnant patients with FS [7].

This case report aims to describe the challenges and outcomes of obstetric management in a pregnant patient with FS, highlighting the implications of sacral neuromodulation use and the need for an individualized approach.

## Case presentation

A 25-year-old woman, in her second pregnancy, presented with a history of preterm cesarean delivery in 2020 due to premature rupture of membranes. At seven weeks' gestation during her current pregnancy, she sought emergency care at Hospital do Servidor Público Estadual (HSPE) for hyperemesis gravidarum and reported a 5 kilogram weight loss over six days. She was hospitalized for hydration and antiemetic therapy.

Her medical history included a previous use of oral progestin contraceptives, which she discontinued to conceive, a past diagnosis of syphilis (serological scar), suspected deep endometriosis, and FS managed through sacral neuromodulation by the urology team of HSPE. Following recommendations from this team, she deactivated the neurostimulator and initiated self-catheterization. After three days of hospitalization, during which her vomiting improved, she was discharged. During this hospitalization, the rarity of her condition was explained and discussed, informed consent was obtained, and a case report was initiated.

In the high-risk prenatal follow-up at 13 weeks gestation, the patient reported persistent urinary difficulties, nocturia (three episodes per night), urinary hesitation, and intermittent catheterization (averaging six times daily). She also experienced elevated morning blood pressure. A first-trimester ultrasound revealed no abnormalities. Prophylactic nitrofurantoin was prescribed until 14 weeks, followed by cephalexin until 34 weeks [8].

At 17 weeks, the patient experienced sciatic nerve pain, potentially caused by neurostimulator displacement. Without medical consultation, she reactivated the device, reporting stimulation in the buttocks rather than the perineal region. Despite experiencing urinary urgency, frequency, and a sensation of incomplete bladder emptying, she denied dysuria or urinary tract infections. The patient performed random blood pressure measurements at home without a medical indication for monitoring. These readings showed elevated values, although she had no prior diagnosis of gestational hypertension

Prednisone was prescribed for pain, and amlodipine was initiated for hypertension. Throughout the pregnancy, she remained on amlodipine monotherapy. Second-trimester Doppler ultrasound and urine cultures revealed no abnormalities. However, despite blood pressure control, pain and headaches persisted.

At 28 weeks, fetal echocardiogram and urine cultures were unremarkable. Vaginal discharge accompanied persistent pain and was treated with clotrimazole. By 31 weeks, worsening blood pressure and sporadic uterine contractions were noted. At 32 weeks and two days, she presented to emergency care with frequent contractions. Preterm labor was suspected, and she received betamethasone for fetal lung maturation and beta-adrenergic therapy. Labor was successfully inhibited, and she was discharged on utrogestan and cephalexin.

At 34 weeks, contractions persisted but were non-rhythmic, and urine cultures remained negative.

At 37 weeks and four days, she was admitted in labor with premature rupture of membranes. The mode of delivery was evaluated, but she experienced secondary arrest of labor progression, leading to a transverse cesarean section and bilateral tubal ligation. A healthy female infant (APGAR 8/9, 3365 g) was delivered without complications

Postpartum, the patient reported neurostimulator misalignment, resulting in lower limb shocks. The device was deactivated, and outpatient spinal imaging was scheduled.

Table 1 provides a chronological overview of the patient’s symptoms, medical decisions, and outcomes throughout pregnancy. It highlights key clinical events, including symptom progression, treatment adjustments, and the impact of SNM management on her condition. The timeline also details interventions for pain, urinary symptoms, hypertension, and labor, culminating in a planned cesarean section.

**Table 1:** Timeline of Symptoms, Medical Decisions, and Outcomes During Pregnancy

Timepoint	Symptoms	Medical Decisions	Outcomes
Confirmation of Pregnancy (Early 1st Trimester)	Hyperemesis gravidarum (5 kg weight loss over 6 days)	Hospitalization for hydration and antiemetic therapy.	Stabilization of condition with hospital discharge.
During the 1st Trimester (up to 14 weeks)	Urinary urgency, nocturia (3 episodes/night), urinary hesitancy, persistent urinary difficulties	Discontinuation of sacral neuromodulation (SNM) at the patient's request and initiation of intermittent catheterization.	Improvement of initial urinary symptoms with intermittent catheterization.
16-17 weeks	Sciatic pain, possible neurostimulator displacement	Reactivation of the neurostimulator without medical consultation due to sciatic pain.	Persistent urinary symptoms, but no dysuria or urinary tract infection (UTI).
18-25 weeks	Persistent sciatic pain and morning hypertension	Prescription of prednisone for pain and amlodipine for hypertension control.	Normalization of blood pressure with methyldopa, but persistent pain.
28 weeks	Persistent pain, vaginal discharge, headaches	Treatment with clotrimazole for vaginal discharge.	No abnormalities found in fetal echocardiogram and urine cultures.
31-32 weeks	Sporadic uterine contractions, elevated blood pressure	Monitoring of blood pressure and contractions, administration of betamethasone for fetal lung maturation, and beta-adrenergic therapy for labor inhibition.	Successful inhibition of preterm labor and stabilization of the condition.
34 weeks	Non-rhythmic contractions, urine without infection	Continuous monitoring, use of prophylactic antibiotics (cephalexin).	Pregnancy without urinary tract infection or major complications.
37 weeks and 4 days	Regular contractions, membrane rupture, onset of labor	Planning for cesarean section and tubal ligation.	Cesarean section performed with birth of a healthy newborn (APGAR 8/9, 3365g).
Postpartum (Immediate)	Discomfort with neurostimulator (shocks in lower limbs)	Deactivation of the neurostimulator and scheduling of spinal imaging.	Postpartum discharge without significant complications.

## Discussion

Pregnancy in women with FS presents unique challenges due to the potential for urinary retention, recurrent UTIs, and associated complications. SNM is a primary therapeutic intervention but carries risks during pregnancy, including the theoretical risk of uterine contractions and device displacement [5].

Studies indicate that deactivating the SNM device can lead to increased urinary retention and UTI risks, potentially resulting in preterm labor [6]. Conversely, maintaining SNM activation has been associated with continued voiding function, suggesting that SNM may offer protective benefits during pregnancy [7]. However, no consensus exists regarding optimal management, necessitating individualized, multidisciplinary care.

The mode of delivery is another contentious issue. While cesarean delivery may reduce the risk of device displacement, it can also contribute to postoperative complications. Vaginal delivery, if deemed safe, may offer benefits in terms of quicker recovery and reduced surgical risks [3]. Further studies are needed to establish clear guidelines on the management of Fowler's syndrome in pregnancy to optimize maternal and fetal outcomes.

The comparative analysis of this case with five other reported instances in the literature reveals both commonalities and distinctive differences in the management of Fowler's syndrome during pregnancy. Ansó et al. [9] highlight the approach of deactivating SNM during pregnancy, with successful reactivation postpartum resulting in a favorable vaginal delivery outcome. This contrasts with Alghazwani et al. [4], where continuous SNM activation without complications is documented, illustrating a different but effective management strategy. Khan et al. [10] demonstrated a successful multidisciplinary approach, albeit with reliance on suprapubic catheterization, culminating in an elective cesarean section. This consistency in multidisciplinary management is echoed by Szymański et al. [11], who maintained SNM use without UTIs, yet opted for a cesarean delivery. Conversely, El-Akri and Peyronnet [12] report unique challenges, notably electrode migration post-delivery, highlighting physiological variability in response to SNM. The choice of delivery method also varied, with most cases opting for cesarean sections to mitigate risks associated with SNM placement and management. This case further diverges in its complex symptomatology, including hypertension and sciatic pain, necessitating a multifaceted therapeutic approach. These comparisons underscore the need for tailored management strategies that consider both the physiological and technological aspects unique to each patient, advocating for a personalized approach in the treatment of Fowler's syndrome during pregnancy. Table 2 presents a comparison of my case report with five other cases from the literature in chronological order, highlighting the similarities, differences, and types of delivery for a better understanding of the management of the pathology.

**Table 2:** Comparison of the Current Case with Published Reports on Pregnancy and Sacral Neuromodulation

Author	Year	Similarities with the Current Case	Differences with the Current Case	Type of Delivery
Ansó et al. [9]	2016	Sacral neuromodulation (SNM) deactivated during pregnancy	Reactivation of SNM postpartum	Successful vaginal delivery
Alghazwani et al. [4]	2020	Effective pregnancy management in Fowler's syndrome; multidisciplinary approach	Continuous SNM activation throughout pregnancy with no adverse effects noted	Not specified
Khan AT, Kinder RB, Hayman RG [10]	2020	Effective pregnancy management in Fowler's syndrome; multidisciplinary approach; SNM deactivated during pregnancy	Utilization of suprapubic catheterization	Elective cesarean section
Szymański JK, Słabuszewska-Jóźwiak A, Jakiel G [11]	2022	No urinary tract infections (UTIs) reported	SNM use maintained throughout pregnancy	Cesarean
El-Akri and Peyronnet [12]	2024	Management without occurrence of UTIs	Activation of SNM during pregnancy; post-delivery electrode migration requiring device revision	Vaginal delivery

## Conclusions

This case underscores the complexities of managing FS during pregnancy, highlighting the necessity of a multidisciplinary and individualized approach. The challenges associated with SNM reactivation, including persistent urinary symptoms and discomfort, emphasize the importance of careful monitoring and patient counseling. Notably, despite performing intermittent self-catheterization without strict aseptic techniques, the patient did not develop urinary tract infections, suggesting that this approach may be a feasible option for symptom management in select cases.

A comparison with previously documented cases reveals significant variability in clinical presentation and management strategies. While some reports describe the successful continuation of SNM throughout pregnancy, others, like the present case, required temporary deactivation due to symptom fluctuations and device-related discomfort. The lack of standardized guidelines on SNM use in pregnancy highlights the need for further research to better elucidate the risks and benefits of these interventions and to establish evidence-based management protocols.
